# Advanced Lung Cancer Inflammation Index as Predictor of All-Cause Mortality in ST-Elevation Myocardial Infarction Patients Undergoing Primary Percutaneous Coronary Intervention

**DOI:** 10.3390/jcm13206059

**Published:** 2024-10-11

**Authors:** Giancarlo Trimarchi, Fausto Pizzino, Alessio Lilli, Alberto Ranieri De Caterina, Augusto Esposito, Stefano Dalmiani, Annamaria Mazzone, Gianluca Di Bella, Sergio Berti, Umberto Paradossi

**Affiliations:** 1 Department of Clinical and Experimental Medicine, University of Messina, 98100 Messina, Italy; giancarlo.trimarchi18@gmail.com (G.T.); gianluca.dibella@unime.it (G.D.B.); 2 Interdisciplinary Center for Health Sciences, Scuola Superiore Sant’Anna, 56127 Pisa, Italy; 3 Cardiology Unit, Heart Centre, Fondazione Gabriele Monasterio—Regione Toscana, 54100 Massa, Italy; lillialessio78@gmail.com (A.L.); a.decaterina@gmail.com (A.R.D.C.); augustoesposito1990@gmail.com (A.E.); annamaria.mazzone@ftgm.it (A.M.); ifcberti@ftgm.it (S.B.); uparadossi@ftgm.it (U.P.); 4 Fondazione Toscana Gabriele Monasterio, Via Moruzzi 1, 56100 Pisa, Italy; dalmiani@ftgm.it

**Keywords:** vanced lung cancer inflammation index, ST-elevation myocardial infarction, primary PCI, neutrophil-to-lymphocyte ratio, inflammation, malnutrition, mortality

## Abstract

**Background:** The advanced lung cancer inflammation index (ALI) is an independent prognostic biomarker used to assess inflammation and nutritional status in various cancers, heart failure, and acute coronary syndromes. This study investigates the prognostic significance of ALI in patients experiencing ST-elevation myocardial infarction (STEMI) treated with primary percutaneous coronary intervention (pPCI), comparing its predictive abilities with the established Neutrophil-Lymphocyte Ratio (NLR). **Methods:** We conducted a retrospective analysis of 1171 patients from the Matrix Registry, encompassing demographic and clinical data for STEMI cases treated with pPCI, and ALI was determined using the formula [serum albumin (g/dL) × body mass index (kg/m^2^)]/NLR at the time of hospital admission. The primary outcome was all-cause mortality. **Results:** Of the 1171 patients, 86 died during the follow-up period. Univariate analysis identified age, female gender, smoking, hypertension, diabetes, prior myocardial infarction (PMI), lower left ventricular ejection fraction (LVEF), and reduced ALI as factors associated with mortality. Multivariate analysis confirmed age (HR: 1.1, 95% CI: 1.05–1.11, *p* < 0.001) and PMI (HR: 2.4, 95% CI: 1.4–4.3, *p* = 0.001) as prominent independent predictors, alongside ALI (HR: 0.95, 95% CI: 0.92–0.97, *p* < 0.001) and LVEF (HR: 0.98, 95% CI: 0.97–0.99, *p* = 0.04). An ALI cut-off of ≤10 indicated a higher mortality risk (HR: 2.3, 95% CI: 1.5–3.7, *p* < 0.001). The area under the curve for ALI (0.732) surpassed that for NLR (0.685), demonstrating ALI’s superior predictive capability. **Conclusions:** ALI is an independent prognostic factor for all-cause mortality in STEMI patients undergoing pPCI, showing greater discriminatory power than NLR, particularly in patients with ALI values ≤ 10, who face a 2.3-fold higher mortality risk.

## 1. Introduction

Acute coronary syndrome (ACS) represents a significant contributor to cardiovascular morbidity and mortality on a global scale. Despite considerable advancements in pharmacological therapies and percutaneous interventions, patients with ACS continue to experience high rates of mortality and recurrence [1]. Specifically, in cases of ST-segment elevation myocardial infarction (STEMI), primary percutaneous coronary intervention (pPCI) is regarded to be the optimal reperfusion strategy [2]. However, even with the timely and effective restoration of coronary blood flow, certain patient populations have an elevated risk of death [3]. The accurate assessment of prognostic risk and the establishment of standardized follow-up protocols are acknowledged as critical strategies for enhancing patient survival outcomes. Identifying high-risk patients through the evaluation of modifiable clinical characteristics is essential for implementing targeted interventions aimed at mitigating risk factors [4]. To this end, recent research endeavors have increasingly focused on various inflammatory indices, recognized as convenient and noninvasive tools for assessing prognostic risk in ACS patients [5]. These indices hold promise not only for risk stratification but also for informing tailored therapeutic approaches that could significantly improve patient prognosis and overall outcomes in the context of acute coronary events [6,7].

Inflammation plays a pivotal role in all stages of atherosclerosis, with various mediators contributing to the intricate inflammatory response [8]. These mediators have been recognized as significant adverse prognostic factors in patients with stable coronary artery disease [9] and also in those with STEMI [5]. Recently, there has been considerable interest in inflammatory parameters derived from a standard complete blood count, as they represent simple, readily available, and cost-effective biomarkers [10,11].

During an acute myocardial infarction, distinct blood cell types display a characteristic temporal pattern: neutrophils typically rise early, peaking within one to three days, followed by increases in monocytes and platelets, while lymphocyte counts decline [12,13,14]. Notably, ratios calculated from these cell types—such as the neutrophil-to-lymphocyte ratio (NLR), the platelet-to-lymphocyte ratio (PLR), and the monocyte-to-lymphocyte ratio (MLR)—have been studied extensively and identified as prognostic indicators in patients with coronary artery disease [5]. Additionally, two novel indices, the systemic immune-inflammation index (SII) and the systemic inflammatory response index (SIRI), have proven useful for concurrently evaluating inflammatory and immune statuses, demonstrating predictive capabilities for adverse outcomes in patients experiencing STEMI [6,15].

On the other side, recent evidence underscores malnutrition as a significant poor prognostic factor in cardiovascular disease [16,17,18]. Unlike many other clinical variables, malnutrition is particularly noteworthy because it is a modifiable risk factor, offering an opportunity for intervention by healthcare professionals. Patients with ACS, particularly the elderly, frequently experience malnutrition due to imbalances in nutrition—either over- or under-nutrition—coupled with a gradual decline in physical and physiological function [19]. Research indicates that both ACS and aging are linked to heightened levels of inflammation, which contribute to malnutrition and unfavorable outcomes, including increased all-cause mortality and major adverse cardiac events [20,21].

In this context, the Geriatric Nutritional Risk Index (GNRI) has emerged as a widely used screening tool for assessing nutritional status, incorporating body mass index (BMI) and serum albumin levels [22]. The GNRI has demonstrated predictive value for adverse outcomes in patients undergoing pPCI for ACS [23]. Similarly, the Controlling Nutritional Status (CONUT) score—a newly proposed method for evaluating nutritional status and detecting undernutrition—utilizes serum albumin, total cholesterol, and lymphocyte counts [24]. This low-cost and comprehensive tool has been shown to predict adverse clinical outcomes in STEMI patients undergoing pPCI [25,26]. Nevertheless, nutritional and inflammatory factors remain largely absent from current ACS risk classification and from prognosis assessments commonly employed in clinical practice.

In this context, the demand for a comprehensive index that simultaneously evaluates inflammation and nutritional status could be effectively addressed by the advanced lung cancer inflammation index (ALI). This index is mathematically defined as the product of BMI and serum albumin levels divided by the NLR [27]. It serves as a dual-purpose metric, encapsulating both nutritional and inflammatory parameters. Originally developed for assessing the prognosis of patients diagnosed with non-small cell lung cancer (NSCLC) [28], the ALI has since gained recognition for its efficacy in predicting adverse clinical outcomes across various cancer types [29]. Recent research underscores the utility of ALI beyond oncology, highlighting its predictive capabilities in other medical conditions, such as acute decompensated heart failure [30] and ACS [31,32], particularly concerning major adverse cardiovascular events (MACEs).

In this perspective, we chose to conduct a retrospective clinical study focused on assessing the predictive role of ALI in all-cause mortality among patients with STEMI undergoing PCI. Additionally, we intended to compare ALI’s predictive value with that of the well-established NLR.

## 2. Materials and Methods

All of the patients included in our study have been extracted from the Matrix Registry. This is a single-center non-interventional registry created to evaluate demographic, clinical, and therapeutic characteristics of all-comer patients presenting to our institution with STEMI and treated with pPCI. The “Heart Hospital” is a third-level hub hospital with 24/7 pPCI capability situated in northeast Tuscany (Italy), serving a population of about 400,000 people. STEMI diagnosis was established using ECG registered in the spoke centers or by the emergency service directly in the territory according to the criteria defined by the current guidelines. All of the patients underwent pPCI with respect to the timing defined by the guidelines [33]. The procedure was performed using a radial arteriosus approach in 52% of cases, while the remaining patients were treated via a femoral approach. All of the patients achieved successful revascularization of the culprit vessel and were treated with guideline-directed medical therapy [33]. Patient follow-up was conducted via telephonic interviews by directly contacting the patient or his/her general practitioner or consulting official mortality registries. In order to investigate the effect of ALI on long-term prognosis, all of the patients who died prior to hospital discharge were excluded from the study.

ALI was calculated using the following formula, as previously described: [serum albumin (g/dL) × BMI (kg/m^2^)]/(NLR). For the calculation of ALI, the BMI, albumin, neutrophil, and lymphocyte values at the time of hospital admission were considered.

We collected data from the Matrix Registry regarding classical cardiovascular risk factors (age, gender, smoking status, diabetes, hypertension, dyslipidemia, and coronary artery disease (CAD) familiar history) and other data associated with poor prognosis, including the following: a history of prior myocardial infarction (MI), the anterior localization of MI, presence of non-infarct-related arteries (No-IRAs) critical stenosis (defined as reduction of ≥50% in the vessel lumen), and the left ventricular ejection fraction (LVEF). The LVEF was calculated within 1 day of hospital admission via transthoracic echocardiography with the modified Simpson’s Biplane Method. All of the patients signed informed consent forms for the collection of their clinical data and inclusion in the registry. The study protocol was approved by our ethical committee. This study was conducted in accordance with Good Clinical Practice principles and the Declaration of Helsinki.

### Statistical Analysis

The continuous variables are presented as means and standard deviation or as medians and interquartile ranges depending on the normal or non-normal distribution of the data. Categorical variables are presented as numbers and percentages. Statistical comparisons between the means were conducted using either the *t*-test or the Mann–Whitney U test, depending on the distribution of the data. The distribution of the data was assessed using the Kolmogorov–Smirnov test. Categorical frequencies were compared using the Chi-square test. Collinearity was evaluated using Spearman’s rho correlation. The association between various variables and the outcome was initially examined using univariate Cox regression analysis, while multivariate analysis was performed to assess the independence of these associations. The comparison of event associations with ALI or NLR was assessed by comparing the receiver operating characteristic (ROC) curves. The optimal cut-off associated with the outcome was determined using the Youden index applied to the ROC curve. Differences in survival were evaluated using Kaplan–Meier curves and the log-rank test. All of the tests were performed as two-tailed, and the alpha error for rejecting the null hypothesis was set to 5%. Calculations were performed using SPSS version 20 (IBM Corp., Armonk, NY, USA) and MedCalc version 14 (MedCalc Software Ltd., Ostend, Belgium).

## 3. Results

We retrospectively revised our database, collecting a total of 2506 patients who were referred for STEMI and treated with pPCI from 2006 to 2018. Unfortunately, 90 patients died before discharge, 467 patients were lost to follow-up, and 778 patients were excluded due to incomplete data needed to calculate the ALI. Consequently, 1335 patients were excluded, leaving 1171 in the final analysis.

The median follow-up was 723 (488–1176) days. During follow-up, 86 patients died, and 1085 survived. Table 1 presents a comparison of the various characteristics between the patients who survived to the time of follow-up (survival group) and those who did not (death group). The average age of the patients who died was significantly higher compared to those who survived (77 ± 11 years vs. 65 ± 12 years, *p*-value < 0.001). Female gender constituted a higher percentage among those who died (34.9% vs. 24.1%, *p* = 0.03). Although mean BMI values fell within the normal range in both groups, patients in the survival group showed significantly higher levels (27.5 ± 8.2 vs. 25.2 ± 3.6, *p* = 0.01), reflecting a more robust physical status. Unexpectedly, smoking was less common in the “Death group” (20.9% vs. 46.3%, *p* < 0.001); this can be explained by the so-called “smoking paradox”, which has been described in previous studies [34]. Hypertension (68.6% vs. 56.2%, *p* = 0.03) and diabetes (29.1% vs. 18.7%, *p* = 0.02) were more prevalent in the death group. Patients with a history of prior MI were also more likely to die (22% vs. 10%, *p* = 0.001). Among the survivors, the median neutrophil count was lower [8 × 10^3^/μL (interquartile range (IQR) 6.2–10.4.) vs. 9.3 × 10^3^/μL (IQR 7–12.1), *p* = 0.001]. This significant difference suggests a more intense inflammatory response or the underlying severity of illness in those who succumbed. In contrast, lymphocyte counts showed higher values in the survival group [1.5 × 10^3^/μL (IQR 1–2) vs. 1.1 × 10^3^/μL (IQR 0.8–1.6), *p* < 0.001]. Albumin levels also differed between the two groups. Survivors had a mean albumin level of 3.6 ± 0.4 g/dL, while patients who died had a lower mean albumin level of 3.3 ± 0.5 g/dL (*p* < 0.001), reflecting a poorer nutritional status or more severe illness. As expected, LVEF was confirmed to be significantly lower in the death group (38.3 ± 11% vs. 45.6 ± 10%, *p* < 0.001), while NLR was higher (8.4 vs. 5.6, *p* < 0.001). Regarding ALI, it was significantly lower in the death group (9.6 vs. 17.7, *p* < 0.001). Dyslipidemia (41.6% vs. 33.7%, *p* = 0.17), familial history of CAD (31.7% vs. 20.9%, *p* = 0.51), anterior MI (42.9% vs. 47.7%, *p* = 0.4), and findings of no-IRA critical stenosis (25.7% vs. 34.8%, *p* = 0.9) did not show significant difference between the two groups.

To demonstrate the prognostic role of ALI in our population and the superiority of this index on the simple NLR, we included all the variables that demonstrated differences among the groups in a Cox regression model. However, since ALI includes the NLR in its calculation, we tested the two variables for collinearity. Spearman rho demonstrated a strong inverse correlation between the two variables (−0.95, *p* < 0.001), subsequently excluding the possibility of including the two variables in the same Cox regression model. Therefore, to identify whether ALI was more associated with the outcome than NLR, we compared the predictive accuracy of the two variables using ROC curves analysis.

**Figure 1:**
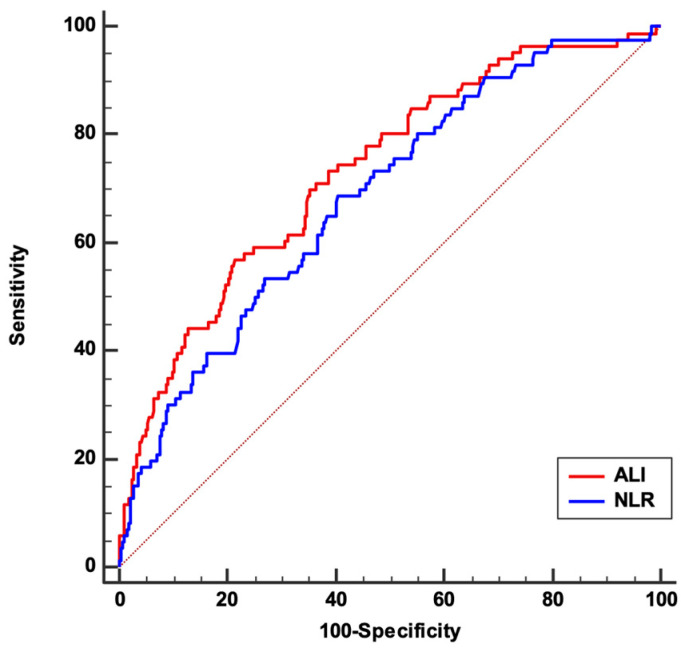
ROC comparison between ALI and NLR, showing greater AUC for ALI vs. NLR (0.732 vs. 0.685; *p* < 0.001) and indicating that ALI had statistically significantly better discriminatory power than NLR for predicting death in this patient cohort. **Abbreviations:** ALI: advanced lung cancer inflammation index; AUC: area under the curve; NLR: neutrophil–lymphocyte ratio; ROC: receiver operating characteristic.

The analysis revealed that the area under the curve (AUC) for ALI was 0.732 (95% CI: 0.706–0.757), while for NLR, it was 0.685 (95% CI: 0.658–0.712). The pairwise comparison of these ROC curves revealed that the difference in AUC between ALI and NLR was 0.0472 (95% CI: 0.0282–0.0662, *p* < 0.001), indicating that ALI had statistically significantly better discriminatory power than NLR in terms of predicting death in this patient cohort, as shown in Figure 1. The AUC of ALI was significantly associated with the outcome (*p* < 0.001), and the Youden index identified an associated criterion of ≤10, with a sensitivity and specificity of 56.98% and 78.62%, respectively. Aiming to test the association to our endpoint of the variables showing significant differences between the two groups, we included the single variables in a univariate Cox regression model.

In the univariate analysis (see Table 2), age was found to be a significant predictor of the outcome (HR: 1.1, 95% CI: 1.07–1.12, *p* < 0.001), indicating that with each additional year of age, the risk increases by 10%. Female gender was associated with a doubled risk of death (HR: 2.0, 95% CI: 1.3–3.2, *p* = 0.002). Smoking confirmed an apparent protective role (HR: 0.3, 95% CI: 0.2–0.5, *p* < 0.001), while hypertension (HR: 1.6, 95% CI: 1.0–2.5, *p* = 0.04) and diabetes (HR: 1.7, 95% CI: 1.0–2.6, *p* = 0.03) were associated with increased risk. Prior MI was also associated with increased risk (HR: 2.1, 95% CI: 1.3–3.5, *p* = 0.004). Lower LVEF was associated with increased risk (HR: 0.94, 95% CI: 0.92–0.96, *p* < 0.001), as was lower ALI (HR: 0.93, 95% CI: 0.90–0.95, *p* < 0.001).

In order to test the independence of the association, all the variables associated with the outcome were included in a multivariate Cox regression model (see Table 2).

In the multivariate analysis (Table 2), female gender was not independently associated with the outcome (HR: 1.4, 95% CI: 0.9–2.3, *p* = 0.1), indicating that when other variables are controlled for, the effect of gender is not statistically significant. The most probable reason for the apparent influence of gender on death in our population is due to the confounding effect of age; in fact, females were significantly older than males (72 ± 12 vs. 64 ± 12 years, *p* < 0.001). Moreover, smoking (HR: 1; 95% CI: 0.6–1.9; *p* = 0.9) and hypertension (HR: 1; 95% CI: 0.6–1.7; *p* = 0.9) lost their significance, suggesting that their univariate associations were confounded by other variables. The same happened for diabetes (HR: 1.4; 95% CI: 0.8–2.2, *p* = 0.2). Age and Prior MI remained strong independent predictors of death (HR: 1.1; 95% CI: 1.05–1.11, *p* < 0.001 and HR: 2.4; 95% CI: 1.4–4.3, *p* = 0.001, respectively), as did LVEF; however, its effect was attenuated (HR: 0.98; 95% CI: 0.97–0.99, *p* = 0.04). It is worth highlighting that ALI remained a significant and independent predictor of death in our cohort of patients (HR: 0.95; 95% CI: 0.92–0.97, *p* < 0.001).

We aimed to include a model using discrete values clustered around the following cut-off points: for LVEF, the value most frequently cited in the literature to identify patients with severe dysfunction and a poorer prognosis, and for ALI, the threshold identified through our ROC curve analysis (<35% and ≤10, respectively). This approach was also chosen to provide a more clinically interpretable representation of the data, as clinicians often find it easier to identify and recall distinct cut-off points rather than deal with progressively increasing risks associated with each incremental change in variable values.

**Figure 2:**
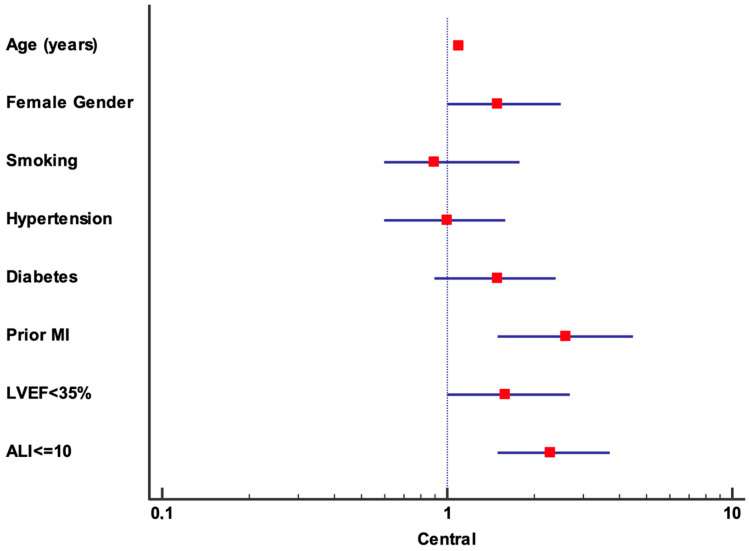
Forrest plot representing HR of multivariate analysis treating LVEF and ALI as discrete variables (with cut-offs at 35% and 10, respectively). **Abbreviations:** ALI: advanced lung cancer inflammation index; HR: hazard ratio; LVEF: left ventricular ejection fraction; MI: myocardial infarction.

When conducting the multivariate analysis treating LVEF and ALI as discrete variables (with cut-offs at 35% and 10, respectively), LVEF (HR: 1.6, 95% CI: 1–2.7, *p* = 0.48) loses its statistical significance. Conversely, prior MI (HR: 2.6, 95% CI: 1.5–4.5, *p* = 0.001) and ALI (HR: 2.3, 95% CI: 1.5–3.7, *p* < 0.001) remain independent predictors of the outcome, with ALI presenting a higher HR compared to when it is considered a continuous variable. However, the loss of predictive power when using LVEF as a discrete variable suggests that, in our population, LVEF likely has a more linear association with risk, extending even at levels higher than 35%. This may also be due to the generally lower baseline LVEF levels in our population, meaning that a severely reduced LVEF does not adequately capture increased risk in this patient group. The results of this second multivariate analysis are shown in Table 3 and in Figure 2.

**Figure 3:**
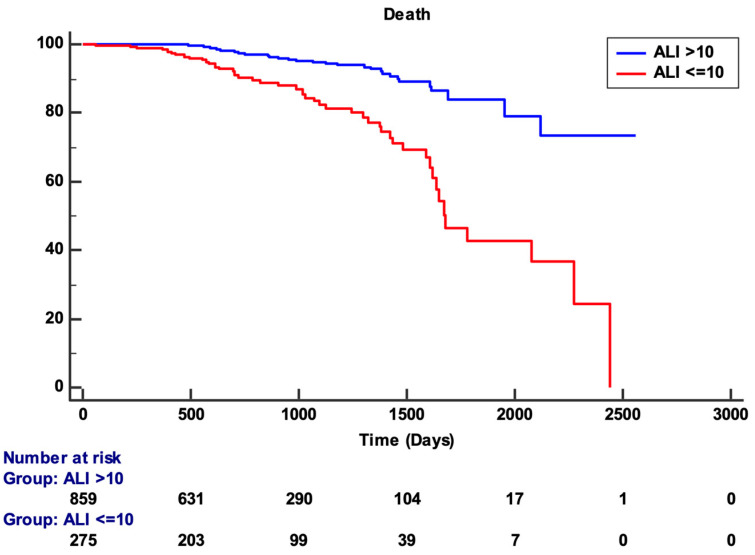
Kaplan–Meier survival curves showing the different survival of STEMI patients according to ALI > 10 and ALI ≤ 10. **Abbreviations:** ALI: advanced lung cancer inflammation index.

To demonstrate the different survival of patients according to ALI, we clustered the population according to the best-associated criterion of the Youden test (ALI ≤ 10) and showed the different values of free-event survival by using Kaplan–Meier Curves (Figure 3). The graph clearly shows a strong difference in survival between the two populations. The difference in survival is significant, as revealed by the log-rank test (*p* < 0.001).

As shown by the Kaplan–Meier curves, the impact of ALI ≤ 10 on survival becomes apparent after 365 days and becomes more pronounced by 500 days. From that point, the survival curves increasingly diverge, with a widening gap. This indicates that the prognostic effect of ALI remains consistent over time, lasting up to at least 1500 days (approximately 4 years). After this point, the survival curve for the ALI ≤ 10 group declines further, reflecting a worsening prognosis for this group. Beyond 1600 days, the precision of the curves decreases due to the relatively low number of patients remaining at risk.

## 4. Discussion

The primary findings of our study are as follows: (1) the ALI serves as an independent prognostic factor for all-cause mortality in patients with STEMI who undergo pPCI; (2) within this specific population, the ALI demonstrates superior prognostic capabilities compared to the simple NLR; and (3) patients with STEMI undergoing pPCI and exhibiting ALI values ≤ 10 present a 2.3-fold greater risk of all-cause mortality compared to those with ALI values > 10.

Inflammation is a critical component in the pathogenesis of atherosclerosis, which is recognized as the underlying mechanism contributing to ACS. This highlights a significant therapeutic target, as illustrated by recent research that advocates for the use of low-dose colchicine (0.5 mg daily) to mitigate risks in patients with atherosclerosis, effectively reducing the incidence of adverse cardiovascular events and recurrent myocardial infarctions [35,36].

Atherosclerosis is characterized as a chronic inflammatory disease stemming from immune–inflammation disorders involving interactions between immune cells (neutrophils and lymphocytes) and vascular cells (endothelial and smooth muscle cells) [37,38]. A study conducted by Shumilah et al. demonstrated that patients with ACS exhibit elevated total white blood cell counts, specifically neutrophils and monocytes, alongside reduced lymphocyte counts compared to healthy individuals [39]. The release of inflammatory mediators increases neutrophil production, enhancing their capacity to phagocytize pathogens. Conversely, lymphopenia during inflammation may be attributed to rising corticosteroid levels and apoptosis induced by inflammatory processes, signifying an active, nonspecific atherosclerotic inflammatory state. Furthermore, the NLR has been recognized as being a reliable indicator of systemic inflammation and has been identified as an independent prognostic marker for ACS outcomes [40,41,42].

Another critical aspect to consider in patients with ACS is malnutrition. Malnutrition, characterized by either over-nutrition or under-nutrition, is prevalent among hospitalized ACS patients, particularly the elderly, and is associated with a poor prognosis [20,43]. An observational study conducted by Raposeiras Roubín et al. revealed that approximately 50% to 60% of patients with ACS were classified as malnourished based on various nutritional assessment scores. These malnourished individuals experienced a higher incidence of MACEs as well as increased all-cause mortality [21].

Although it has been noted that nutritional status can significantly influence the prognosis of patients with ACS, routine clinical assessments of nutritional markers to predict their outcomes remain absent. Malnutrition, akin to inflammation, is especially significant as it is a modifiable risk factor, presenting an opportunity for healthcare professionals to implement interventions. In this context, the ALI, calculated as BMI × albumin/NLR, emerges as a simple and cost-effective marker. Indeed, ALI effectively condenses the validated inflammatory status index, represented by NLR, with a simple and reliable nutritional index, the GNRI, calculated on the basis of body weight and serum albumin levels. This allows the two indices to be integrated, providing a more comprehensive evaluation of the patients and enhancing their prognostic value. Initially utilized as a prognostic marker in patients with NSCLC, subsequent studies have demonstrated ALI’s predictive value for various cancers, including gastric and colorectal cancer [44]. Recent evidence has further substantiated ALI’s role as an independent predictor of long-term mortality in elderly patients with heart failure [27].

In our study, we explore the role of ALI in terms of predicting cardiovascular mortality among patients with STEMI undergoing pPCI. To calculate ALI, we considered values obtained at the time of hospital admission. This approach aligns with methodologies employed in other studies addressing similar topics [31,32].

Zhao et al. recently examined the predictive value of ALI for MACEs in 586 elderly patients with ACS, demonstrating its independent predictive value for long-term MACEs in this demographic, which was superior to that of NLR and other indices such as the prognostic nutritional index (PNI) and the GNRI [32].

Similar findings were reported by Wang et al., who assessed a cohort of 1624 ACS patients undergoing PCI and found that a low ALI was associated with poor prognosis, functioning as an independent risk factor for MACEs [45]. Even in the subset of NSTEMI patients, Konuş et al. recently demonstrated that low ALI values predict high SYNTAX scores; an angiographic tool was used to assess the extent and severity of CADs [46].

Our study focuses specifically on STEMI population, representing a group who are at a heightened risk of mortality despite the timely restoration of coronary blood flow [47]. In this context, identifying the most vulnerable populations through appropriate risk stratification is crucial. In our findings, an ALI cut-off of 10 was determined to best predict all-cause mortality in the STEMI cohort receiving pPCI. Notably, patients with an ALI ≤ 10 had a 2.3-fold increased risk of all-cause mortality compared to those with ALI > 10. This aligns with the work of Gong et al., who found that in 217 consecutive patients with acute myocardial infarction complicated by cardiogenic shock, an ALI cut-off of ≤12.69 was an independent predictor for 30-day mortality (hazard ratio [HR]: 3.327; 95% CI: 2.053, 5.389; *p* < 0.001) and 30-day MACEs (HR: 2.250; 95% CI: 1.553, 3.260; *p* < 0.001) [31].

Additionally, we demonstrated that the predictive capability of the ALI for all-cause mortality notably exceeds that of the more commonly studied NLR. A recent meta-analysis conducted by Banahene et al., which analyzed 37 studies involving patients with both STEMI and non-ST-elevation myocardial infarction (NSTEMI), highlighted the heightened risk of MACEs and all-cause mortality associated with elevated NLR values [48]. The odds ratio for MACEs was found to be 1.86 (95% CI 1.53–2.28, *p* < 0.01), while the odds ratio for all-cause mortality was 2.29 (95% CI 1.94–2.70, *p* < 0.01) [48].

The ALI’s superiority over the NLR can be attributed to its comprehensive evaluation of both inflammatory and nutritional status, providing a more holistic assessment of the patient’s condition. In our multivariate Cox regression analysis, where LVEF and ALI were treated as discrete variables with thresholds set at 35% and 10, respectively, ALI was identified as an independent predictor of all-cause mortality, ranking second in hazard ratio after prior myocardial infarction history. Otherwise, we emphasize that the use of ALI for evaluating the prognosis of patients after STEMI should complement, rather than replace, other well-established scores, such as the thrombolysis in myocardial infarction (TIMI) risk score or the Global Registry of Acute Coronary Events (GRACE) risk score.

Based on these considerations, the ALI proves to be a valuable and accessible tool that serves as a reliable index for assessing risk in STEMI patients undergoing pPCI. Drawing on an integrated assessment of inflammatory and nutritional status, the ALI facilitates the identification of patients who are at heightened risk. This capability is instrumental for healthcare providers, as it enables them to make data-driven clinical decisions that could significantly enhance patient outcomes. By using the ALI, clinicians gain a nuanced understanding of the multifaceted factors influencing patient prognosis.

In summary, integrating the ALI into clinical practice has the potential to improve the accuracy of risk assessments and enable more tailored treatments for STEMI patients undergoing pPCI, addressing both inflammation and malnutrition aspects.

## 5. Limitations

This study has several limitations. Firstly, its retrospective design may introduce bias, affecting the reliability of the findings. The all-comers design, though reflective of real-life scenarios, introduces variability that may have influenced the outcomes. Furthermore, although ALI has a strong predictive value, it is inherently variable. We measured ALI based on values registered at the time of admission without accounting for potential variations during follow-up. Despite demonstrating a strong prognostic value for death (ALI ≤ 10), its sensitivity and specificity are relatively low at 57% and 79%, respectively. Additionally, we lack data on the possible revascularization of other vessels. However, the prevalence of critical non-IRA stenosis does not differ significantly between the survival and death groups. An additional limitation of our study is the substantial loss of patients from the initial population due to missing values required for the computation of ALI (778 out of 2506). We acknowledge that this could introduce selection bias, potentially affecting our results. However, no identifiable common patterns were observed in the excluded population and no significant differences were present between the excluded population and the patients included in the final analysis of the study, limiting the likelihood of significant data distortion.

Another limitation of this study is its monocentric nature, relying on data from a single center. Multicenter studies could validate the findings by involving wider patient populations, enhancing the generalizability and applicability of ALI. This broader approach would ensure the conclusions are more universally relevant and reliable across various environments. Lastly, 22% of the patients in the death group had a prior MI compared to only 10% in the survival group. While prior MI is a strong predictor of death alongside ALI, our analysis shows their independent roles in predicting mortality.

## 6. Conclusions

In conclusion, our study highlights the significant role of the ALI as an independent prognostic factor for all-cause mortality in patients suffering from STEMI undergoing pPCI. The findings underscore ALI’s superior prognostic capability compared to the NLR, further emphasizing its utility in risk stratification within this specific patient cohort. The superiority of the ALI compared to the NLR lies in its ability to provide an integrated assessment of both nutritional and inflammatory states, whereas the NLR only considers the inflammatory state and neglects the nutritional aspect.

The statistic that patients with an ALI value of ≤10 face a 2.3-fold increased risk of mortality compared to those with values exceeding 10 aligns with the necessity for clinicians to consider ALI as part of their assessment strategy. These results not only advocate for the incorporation of the ALI into clinical practice for enhanced prognostic accuracy but also signal the need for further research to elucidate the underlying mechanisms by which the ALI influences patient outcomes post-STEMI. Ultimately, our findings contribute to a growing body of evidence supporting the use of inflammatory and nutritional markers in cardiovascular risk assessment, potentially guiding more tailored therapeutic approaches that can improve survival rates in this vulnerable population.

## Figures and Tables

**Table 1:** Anamnestic, clinical, and laboratory characteristics of patients with STEMI at hospital admission, categorized by survival or death at all-cause mortality follow-up.

	Total Population, N = 1171	Survival Group, N = 1085	Death Group, N = 86	*p*
Age (years)	65 ± 12	65 ± 12	77 ± 11	<0.001
Gender (female)	311 (24.2%)	261 (24.1%)	30 (34.9%)	0.03
BMI, kg/m^2^	27.4 ± 8.2	27.5 ± 8.5	25.2 ± 3.6	0.01
Smoking	520 (44.4%)	502 (46.3%)	18 (20.9%)	<0.001
Hypertension	669 (57.1%)	610 (56.2%)	59 (68.6%)	0.03
Diabetes	228 (19.5%)	203 (18.7%)	25 (29.1%)	0.02
Dyslipidemia	481 (41.1%	452 (41.6%)	29 (33.7%)	0.17
CAD familial history	362 (30.9%)	344 (31.7%)	18 (20.9%)	0.51
Anterior MI	507 (43.3%)	466 (42.9%)	41 (47.7%)	0.4
No-IRA critical stenosis	309 (26.4%)	279 (25.7%)	30 (34.8%)	0.9
Prior MI	129 (11%)	110 (10%)	19 (22%)	0.001
Neutrophils (10^3^/μL)	8 (6.2–10.4)	8 (6.1–10.3)	9.3 (7–12.1)	0.001
Lymphocytes (10^3^/μL)	1.5 (1–2)	1.5 (1–2)	1.1 (0.8–1.6)	<0.001
Albumin, g/dL	3.6 ± 0.4	3.6 ± 0.4	3.3 ± 0.5	<0.001
LVEF (%)	45 ± 10	45.6 ± 9	38.3 ± 11	<0.001
NLR	5.7 (3.5–9)	5.6 (3.5–8.5)	8.4 (5.5–14.1)	<0.001
ALI	17 (10–27)	17.7(11–27.8)	9.6 (5.7–15.8)	<0.001

**Abbreviations:** ALI: advanced lung cancer inflammation index; BMI: body mass index; CAD: coronary artery disease; IRA: infarct-related artery; LVEF: left ventricular ejection fraction; MI: myocardial infarction; NLR: neutrophil–lymphocyte ratio.

**Table 2:** Cox regression analysis in the univariate and multivariate model.

Univariate			Multivariate		
	HR (95% CI)	*p*		HR (95% CI)	*p*
Age (years)	1.1 (1.07–1.12)	<0.001	Age (years)	1.1 (1.05–1.11)	<0.001
Gender (female)	2 (1.3–3.2)	0.002	Gender (female)	1.4 (0.9–2.3)	0.1
Smoking	0.3 (0.2–0.5)	<0.001	Smoking	1 (0.6–1.9)	0.9
Hypertension	1.6 (1–2.5)	0.04	Hypertension	1 (0.6–1.7)	0.9
Diabetes	1.7 (1–2.6)	0.03	Diabetes	1.4 (0.8–2.2)	0.2
Prior MI	2.1 (1.3–3.5)	0.004	Prior MI	2.4 (1.4–4.2)	0.001
LVEF	0.94 (0.92–0.96)	<0.001	LVEF	0.97 (0.95–0.99)	0.01
ALI	0.93 (0.90–0.95)	<0.001	ALI	0.95 (0.92–0.97)	<0.001

**Abbreviations:** ALI: advanced lung cancer inflammation index; CI: confidence interval; HR: hazard ratio; LVEF: left ventricular ejection fraction; MI: myocardial infarction.

**Table 3:** Cox regression analysis in the multivariate model treating LVEF and ALI as discrete variables (with cut-offs at 35% and 10, respectively).

Multivariate Analysis		
	HR (95% CI)	*p*
Age (years)	1.1 (1.1–1.11)	<0.001
Gender (female)	1.5 (1–2.5)	0.1
Smoking	0.9 (0.6–1.8)	0.9
Hypertension	1 (0.6–1.6)	0.9
Diabetes	1.5 (0.9–2.4)	0.2
Prior MI	2.6 (1.5–4.5)	0.001
LVEF < 35%	1.6 (1–2.7)	0.48
ALI ≤ 10	2.3 (1.5–3.7)	<0.001

**Abbreviations:** ALI: advanced lung cancer inflammation index; CI: confidence interval; HR: hazard ratio; LVEF: left ventricular ejection fraction; MI: myocardial infarction.

## Data Availability

The data that support the findings of this study may be made available from the corresponding author upon reasonable request.
